# Gut Microbiota and COVID-19: Potential Implications for Disease Severity

**DOI:** 10.3390/pathogens11091050

**Published:** 2022-09-15

**Authors:** Giulia Rocchi, Marta Giovanetti, Francesca Benedetti, Alessandra Borsetti, Giancarlo Ceccarelli, Davide Zella, Annamaria Altomare, Massimo Ciccozzi, Michele Pier Luca Guarino

**Affiliations:** 1 Department of Science and Engineering for Human and the Environment, University of Campus Bio-Medico, 00128 Rome, Italy; 2 Laboratorio de Flavivirus, lnstituto Oswaldo Cruz/Fundação Oswaldo Cruz, Rio de Janeiro 21040-360, Brazil; 3 Department of Science and Technology for Humans and the Environment, University of Campus Bio-Medico, 00128 Rome, Italy; 4 Institute of Human Virology and Global Virus Network Center, Department of Biochemistry and Molecular Biology, University of Maryland School of Medicine, Baltimore, MD 21201, USA; 5 National HIV/AIDS Research Center, Istituto Superiore di Sanità, Viale Regina Elena, 299, 00161 Rome, Italy; 6 Department of Public Health and Infectious Diseases, Sapienza University of Rome, 00161 Rome, Italy; 7 Unit of Digestive Disease, Campus Bio-Medico University, 00128 Rome, Italy; 8 Medical Statistic and Molecular Epidemiology Unit, University of Biomedical Campus, 00128 Rome, Italy

**Keywords:** SARS-CoV-2, COVID-19, microbiome, gastrointestinal symptom, nutrition

## Abstract

The SARS-CoV-2 pandemic resulted in an unprecedented global crisis. SARS-CoV-2 primarily causes lung infection trough the binding of the virus with the ACE-2 cell receptor located on the surface of the alveolar epithelial cells. Notably, ACE-2 cell receptors are also expressed in the epithelial cells of the intestinal tract (GI). Recent data showed that the microbial communities of the GI might act as local and systematic inflammatory modulators. Gastrointestinal symptoms, including diarrhea, are frequently observed in infected individuals, and recent released data indicate that SARS-CoV-2 may also spread by fecal–oral transmission. Moreover, the gut microbiota’s ecosystem can regulate and be regulated by invading pathogens, including viruses, facilitating an effective immune response, which in turn results in less severe diseases. In this regard, increased SARS-CoV-2 mortality and morbidities appear to be frequently observed in elderly immunocompromised patients and in people with essential health problems, such as diabetes, who, indeed, tend to have a less diverse gut microbiota (dysbiosis). Therefore, it is important to understand how the interaction between the gut microbiota and SARS-CoV-2 might shape the intensity of the infection and different clinical outcomes. Here, we provide insights into the current knowledge of dysbiosis during SARS-CoV-2 infection and methods that may be used to re-establish a more correct microbiota composition.

## 1. Introduction

Coronavirus disease 2019 (COVID-19), related to the severe acute respiratory syndrome coronavirus 2 (SARS-CoV-2), resulted in a catastrophic global crisis [1]. Although primarily mild respiratory symptoms, including the common cold, are usually observed in patients, severe cases are usually associated with a cytokine storm that may lead to multi-organ dysfunction [1,2]. Many organs can indeed be affected by the COVID-19 infection, including the lung, kidneys, heart, liver, the central nervous system, and the gastrointestinal tract (GI) [3]. Recent data have revealed that resident microbial communities of the GI might be able to act as local and systematic inflammatory modulators, providing evidence of a communication axis between the gut microbiota and several organs in the human body [4].

Due to this important role, the gut microbiota has often been named ‘the second brain’. Indeed, the recent SARS-CoV-2 pandemic has reinforced the notion that the composition of the gut microbiota might contribute to shaping the disease progression and clinical outcome. In particular, recently, evidence suggested that the microbiota might be significantly altered (dysbiosis) in infected patients, particularly in those presenting with evidence of a long COVID condition (post-associated COVID sequelae, PACS). In this regard, gastrointestinal symptoms and the diagnosis of gut dysbiosis have been observed among infected SARS-CoV-2 patients and are more prevalent among those who present with long-term symptoms [5]. Furthermore, preliminary studies reported that, in these patients, the fecal microbiota was significantly modified as compared with healthy subjects, presenting an enrichment of opportunistic pathogens and depletion of beneficial bacteria [6]. In COVID-19 patients, the presence of GI symptoms significantly increased the deterioration of their clinical symptoms [5,6]. Here, we review the current knowledge of dysbiosis during SARS-CoV-2 infection and the methods that may be used to re-establish a more correct microbiota composition. 

## 2. SARS-CoV-2 Pandemic

By the end of December 2019, the Chinese health authorities reported a cluster of cases of pneumonia with an unknown etiology in the city of Wuhan, Hubei Province, China [7]. On 9 January 2020, the Chinese CDC identified the novel coronavirus later described as severe acute respiratory syndrome coronavirus 2 (SARS-CoV-2) [7,8,9], a single-stranded RNA virus, belonging to the *Coronaviridae* family, with a whole genome of 29,881 bp [10].

Human-to-human transmission was reported in late January 2020, and the World Health Organization (WHO) declared a state of pandemic on 11 March 2020 [11].

Infected patients presented with a wide range of symptoms, including fever, cough, shortness of breath or difficulty breathing, headache, loss of taste or smell, and fatigue [12,13]. Additionally, some patients also presented with gastrointestinal disorders such as diarrhea, nausea, and vomiting [14,15,16].

During the course of the pandemic, mutations have produced many strains (variants/lineages) with varying degrees of infectivity and virulence [16,17]. Some SARS-CoV-2 variants are considered to be variants of concern (VOCs), including Alpha, Beta, Gamma, Delta, and Omicron [18]. These variants are characterized by the presence of a constellation of mutations in the receptor binding domain (RBD) of the S-protein and seem to be associated with increased transmissibility, infection severity, and the ability to escape from neutralizing antibodies [19].

### Inflammation and COVID-19 

The innate and adaptive immune systems are both implicated in the pathogenesis of COVID-19. During viral infection, in some patients, altered T-cells, monocytes, neutrophils, and secondary messengers of inflammation play key roles in the progression of the disease, which may manifest as a mild common cold or, alternatively, lead to severe and/or fatal outcomes of bilateral pneumonia. 

It is well known that the receptor gateway of SARS-CoV-2 is represented by a hydrophobic pocket of the extracellular catalytic domain of the angiotensin-converting enzyme 2 (ACE2), located on the membrane surface of the host cell and expressed in the vascular endothelial cells and pulmonary and intestinal epithelium, as well as in the heart, brain, and kidneys [20]. Viral entry is initiated by contact between the S1 subunit of the RBD and the ACE2 receptor, followed by the fusion of the S2 subunit to the cell membrane. Upon entry by endocytosis, the viral RNA is released into the cytoplasmic environment. By exploiting the machinery of the human host, the viral RNA is then translated, leading to the production of specific viral polyproteins and subsequent formation of the NPS complex and replicase-transcriptase, allowing the virus to replicate its genome and synthesize the S, E, M, and N structural proteins [20]. Concomitantly with the onset of SARS-CoV-2 viral infection, the downregulation of the ACE2 receptor action occurs, with a subsequent local increase in angiotensin II levels and, thus, the dysregulation of the renin–angiotensin–aldosterone system (RAAS). 

Moreover, in the advanced stage of the disease, what occurs in some patients is cytokine release syndrome (CRS), which is responsible for the onset of severe complications associated with the severity of SARS-CoV infection, such as acute respiratory distress syndrome (ARDS) and multi-organ damage [21]. Infected cells trigger an uncontrolled immune response as a result of the recruitment of T-lymphocytes, monocytes, and neutrophils and the release of inflammatory messengers, providing a molecular explanation for the evolution of the COVID-19 infection. A ‘cytokine storm’ is thus observed with high levels of TNF-α, IFN-γ, IL-1β, IL-1, IL-6, IL-8, IL-12, interferon-gamma-inducible protein 10 (IP10), macrophage inflammatory protein 1A (MIP1A), monocyte chemotactic protein 11 (MCP1) and granulocyte-macrophage colony-stimulating factor (GM-CSF) [22,23]. In particular, it has been documented that, in severe phenotypes of COVID-19 infection, there is a dramatic elevation of circulating levels of IL-6 and TNF-α. Local and systemic inflammation induces increased vascular permeability and ischemic damage, thrombosis or thrombo-inflammation, pulmonary edema, and multi-organ complications [24,25]. It follows that, in clinically at-risk individuals and in the presence of pre-existing comorbidities, the complexity of these events may even result in patient death. 

## 3. The Gastrointestinal Tract and COVID-19 Infection

SARS-CoV-2 virus infection is known to cause respiratory symptoms, but its presence in the gastrointestinal tract leads to the exacerbation of various gastrointestinal (GI) manifestations. Although the pathogenesis of short- and long-term GI abnormalities due to coronavirus infection is not yet fully understood, it should probably be considered multifactorial due to the various mechanisms of action of the virus in the intestine, including direct viral cytotoxicity mediated by ACE2 on the intestinal mucosa, inflammation caused by elevated levels of inflammatory cytokines, the alteration of the intestinal microbial ecosystem, and vascular abnormalities [26].

### 3.1. Gastrointestinal Clinical Manifestations and Abnormalities 

Following acute SARS-CoV-2 infection, persistent symptomatology, referred to as ‘long COVID’ or ‘post-acute COVID-19 syndrome’ (PACS), is often reported in a subgroup of convalescent individuals, with the most common manifestations being systemic, cardio-respiratory, neuropsychiatric, and gastrointestinal in nature [27]. Indeed, there is a high prevalence of GI symptoms in a subgroup of individuals, and it is estimated that 73% of patients have diarrhea and abdominal pain/discomfort during the course of illness [26,28]. According to the results obtained from Tariq et al.’s meta-analysis of 78 studies including a total of 12,797 COVID-19 individuals, 1 in 5 patients reported a weighted prevalence of anorexia/loss of appetite (22.3%), diarrhea (12.4%), nausea and/or vomiting (9%), and abdominal pain (6.2%) [29]. The study also reported that the mortality rate among patients with gastrointestinal symptoms was similar to the overall mortality rate [29]. Moreover, another meta-analysis of 60 studies documented the persistence of the same gastrointestinal symptoms following SARS-CoV-2 infection in 4243 patients [30]. Interestingly, according to a prospective cohort involving a total of 1783 veterans of SARS-CoV-2 infection, the persistence of heartburn (16%), constipation (11%), diarrhea (10%), abdominal pain (9%), nausea and/or vomiting (7%) were reported by 220 individuals even 6 months after SARS-CoV-2 infection [31]. In addition, cases of acute mesenteric ischemia and portal vein thrombosis have also been described as complications of the viral infection [32].

The underlying mechanisms responsible for the exacerbation of gastrointestinal abnormalities are still not fully understood. However, the presence of viral RNA genome detected in the stool samples of 86%–100% of patients over more than 30 days following the onset of infection [33], as well as positive staining of the viral nucleocapsid protein in the cytoplasm of gastric, duodenal, and rectal epithelium in endoscopic samples [28], allow us to infer a reasonable infectious capacity and pathogenic effect of the virus at the enteric level. To confirm this, some investigators have demonstrated efficient acute viral replication in the colorectal adenocarcinoma cell line Caco-2 [34] and in the mucosa of the small intestine [28]. In addition, numerous infiltrating plasma cells and T-lymphocytes with interstitial edema were observed in the lamina propria of the stomach, duodenum, and rectum [35], as was an enhanced ability of SARS-CoV-2 to induce the increased production of INF-α, INF-β, and INF-λ in ex-vivo human intestinal tissues [36]. Therefore, although no obvious damage to the intestinal tissues has been found, it is conceivable that the virus is involved in indirectly damaging the intestinal epithelial cells and generating a specific immune inflammatory environment so as to influence the clinical course and manifestations, ranging from abdominal pain/cramps and secretory diarrhea to increased intestinal permeability.

Structural protein E and viroporin Orf3a of the K+ ion channel, in the presence of SARS-CoV-2 virus, have been shown to act on the intestinal epithelium, respectively, by binding the thigh-junction-associated protein PALS1 that connects the epithelial cells [37] and increasing K+ ion leakage from the enterocytes [38], causing the disruption of the barrier integrity, intracellular ionic imbalances, and colitis. These observations could partially explain the increased levels of inflammatory cytokines found in the serum of COVID-19 patients with diarrhea [39]. Therefore, in light of these data, it is reasonable to hypothesize that intestinal dysfunction caused by SARS-CoV-2 contributes to the development of a local pro-inflammatory environment, in addition to promoting the translocation of intestinal bacterial agents to the systemic bloodstream, often characterized by marked alterations, as observed in COVID-19 and PACS patients [40], thus increasing extraintestinal immune inflammatory damage by inflammasome activation and susceptibility to the development of uncontrolled cytokine release syndrome and multi-organ damage. 

An additional etiopathogenic hypothesis of the presence of gastrointestinal changes in COVID1-19 patients could be attributable to the ACE2 receptor, which is highly expressed in the intestinal epithelial cells. At the intestinal level, ACE2 is an important regulator of Na^+^ and the neutral amino acid co-transporter B^0^AT1, as well as intestinal microbial homeostasis, innate immunity, intestinal inflammation, and colitis susceptibility [41]. Hashimoto et al. have shown that the absence of the B^0^AT1 transporter on the apical surfaces of enterocytes in ACE2 mutant mice, in addition to causing a drastic reduction in Na^+^ and amino acid uptake, markedly increased the mice’s susceptibility to epithelial inflammation and immunity through the attenuation of the mTOR pathway and altered expression of antimicrobial peptides (AMPs) by the Paneth cells, as well as increased autophagy [42]. This mTOR–autophagy molecular crosstalk provides mechanistic insights into how SARS-CoV-2 adhesion to the ACE2/B^0^AT1 complex could potentially lead to altered enterocyte viability, epithelial barrier dysfunction, and chronic gastrointestinal dysfunction. The dysfunction of the absorptive mechanisms of tryptophan, phenylalanine, glutamine, and leucine can trigger an ionic imbalance in the intestinal lumen that can easily result in persistent diarrhea and inflammation, in addition to altering the ecology of the bacterial flora. 

However, these associations are based on indirect observations. To date, the molecular mechanisms underlying the gastrointestinal abnormalities caused by the novel coronavirus in a subset of individuals are still under-explored. Recent reports support the hypothesis of interindividual variability in the ACE2 expression and increased susceptibility to systemic cytokine storm mediated by microbiological factors, which are attributable to differences in the structure and function of the gut microbiota. Future studies are needed in order to understand the kinetics and magnitude of these effects on the clinical outcomes following SARS-CoV-2 infection according to the extrapulmonary mechanisms described above.

### 3.2. The Gut Microbiome and Its Role in COVID-19 Disease

The GI tract harbors a large and diverse microbial community that is extremely active due to its close mutual cooperation with the host, which is important for ensuring metabolic homeostasis and immune maturation. Indeed, the continuous and functional interaction between a “healthy” microbiome—through surface antigens and microbial metabolites—and the host organism provides the essential molecular signals for the fine-tuning of systemic immune responses in different parts of the body, including the respiratory system, and protection from disease. In contrast, a condition of dysbiosis of the gut microbiome may influence immune phenotypes and contribute to interindividual variation in immune inflammatory responses, as well as the development of diseases, including respiratory diseases. 

Preclinical studies have observed an increased susceptibility to influenza virus infection in the lungs in the presence of antibiotic-induced dysbiosis [43] and a reduced clearance of lung infections in germ-free mice [44]. In contrast, studies based on probiotic administration in randomized clinical trials have documented the positive, immunomodulating effect of a “healthy” gut microbial profile on respiratory tract infections by regulating immune cell activation and production of inflammation chemical messengers [45,46,47]. These data confirm the presence of a vital interaction between the gut microbiome and the respiratory system, suggesting that the gut–lung link should be taken into serious consideration in the field of infectious diseases, such as COVID-19.

Not surprisingly, the detection of the SARS-CoV-2 viral genome in the gastrointestinal tract in infected patients and its interaction with the gut microbial ecology have received considerable attention (Table 1). Interesting observations have documented a marked alteration of the gut microbiota in COVID-19 patients compared with control groups, characterized by a reduction in bacterial diversity and substantial compositional differences in the bacterial phyla and genera/species, associated with a marked depletion in commensal bacteria, such as *Bacteroides, Roseburia Faecalibacterium prausnitzii, Eubacterium rectale* and *Bifidobacterium spp, Ruminococcus*, and beneficial *Lachnospiraceae*, as well as the notable enrichment with, for example, *Streptococcus, Rothia, Veillonella, Clostridium hathawayi, Actinomyces viscosus*, and *Bacteroides nordii* [48,49,50,51,52], opportunistic pathogens known to cause bacteremia and bacterial infections in the host [53]. These changes persisted in a subgroup of patients even after 30 days following viral clearance [50]. Other reports have described an enrichment with the opportunistic pathogens *Collinsella aerofaciens, Collinsella tanakaei, Streptococcus infantis*, and *Morganella morganii* in stool specimens of patients with another SARS-CoV-2 viral load, while stool samples of patients with zero or low infectivity had higher levels of bacterial species that are beneficial to host immunity and involved in SCFA production, such as *Parabacteroides, Bacteroides*, and *Lachnospiraeae* [54].

Although it is not possible, using these exploratory studies, to attribute a causal role to these bacteria in the pathogenesis of SARS-CoV-2 infection, these preliminary data underscore the potential role of the gut microbiome as a contributing factor in increasing an individual’s susceptibility to the severe COVID-19 disease course and exacerbating systemic hyperinflammation. The finding of a positive correlation between the enrichment with opportunistic pathogens and higher levels of C-reactive protein (CRP), lactate dehydrogenase, aspartate aminotransferase, and gamma-glutamyl transferase, as well as a lower CD8+ T-cell lymphocyte count and an abnormal increase in circulating levels of TNF- α, IL-6, Il-10, IL-18, and INF-γ [48,49,50,52,55,56], suggests these effects on the clinical outcomes of the disease. In addition, it is useful to consider these additional effects in cases where host immune homeostasis is compromised due to pre-existing comorbidities, which may result in a condition of dysbiosis and chronic inflammatory state at baseline, with an increased risk of developing an extrapulmonary inflammatory state due to cytokine release syndrome. 

To test the hypothesis suggesting that specific microbiome signatures may influence the progression of SARS-CoV-2 infection, several authors performed correlation studies examining the link between identified microbiological features and disease severity. Interestingly, they obtained a negative correlation between disease severity and the abundance of seven bacterial taxa, particularly *F. prausnitzii* and *Alistipes onderdonkii*, associated with the tryptophan metabolism and maintenance of immune homeostasis [53]. Faecalibacterium prausnitzii is considered an important biomarker of health, and it is known that its presence is mostly reduced by Western diets, while it is increased by the Mediterranean diet [57]. Interestingly, preliminary studies have shown that areas with a reduced adherence to the Mediterranean diet within the same country are associated with increased SARS-CoV-2-related mortality rates [58], suggesting that strategies aimed at lifestyle and dietary modifications can help to positively modulate the gut microbiome and play a preventive role in SARS-CoV-2 viral infection.

On the other hand, *Coprobacillus, Clostridium ramosum, C. Hathaway*, and *Erysipelotrichaceae*, which are strongly correlated with diarrhea, inflammation, and severity in IBD [59], showed the best positive correlation with COVID-19 disease severity [51]. The genus *Coprobacillus* was also strongly correlated with the severity of SARS-CoV-2 infection [51]. The latter has been recognized for its strong upregulation of ACE2 expression in the murine colon [60]. *Bacteroides dorei, Bacteroides thetaiotaomicron, Bacteroides massiliensis*, and *Bacteroides ovatus*, reported to reduce the expression of the ACE2-converting enzyme in the murine intestine, were found to be inversely correlated with the viral load in patients’ stool throughout the hospitalization period [51]. According to some authors, the overexpression of ACE2 promotes SARS-CoV-2 viral infection. Therefore, these observations could provide mechanistic insights into how ACE2 expression may be modulated by the gut microbiota and be attributable to differences in its structure and function [61,62,63]. Understanding this ACE2/microbiota interaction could explain the interindividual variability in the severity of SARS-CoV-2 infection and allow us to hypothesize interventions aimed at regulating the ACE2 expression by shaping the gut microbiota, bacterial metabolomics, and host immunity.

A potential additional mechanism through which the gut microbiota may help to modulate the severity of SARS-CoV-2 infection is through the actions of its bacterial metabolites. In this regard, SCFAs represent the classic example demonstrating how bacterial-derived molecules contribute to immune homeostasis at the systemic level [64] and aid in the understanding of disease and infection. SCFAs, such as acetate, propionate, and butyrate, are important metabolites obtained from the anaerobic fermentative processes of dietary fiber in the colon enacted by the gut microbiota. These SCFAs, particularly butyrate, in addition to contributing to the trophism of the colonocytes themselves, help to keep the intestinal epithelial barrier intact. In addition, they have recognized anti-inflammatory and immunoregulatory properties at the systemic level, inhibiting histone deacetylase activity and the activation of the nuclear factor (NF)-kB signaling pathway, and interacting directly with G-protein-coupled receptors (GPR41–43, or FFAR3–2) expressed on various tissues and immune cells [65]. Finally, they stimulate the Treg cells, which play a central role in suppressing inflammatory responses.

However, the typical dysbiosis profile of the microbiota observed in COVID-19 disease, with the depletion of SCFAs-producing bacterial species and overrepresentation of known opportunistic pathogens, causes an imbalance in all the processes described. Metabolomic studies of animal models observed changes in SCFA production in the presence of experimental influenza and SARS-CoV-2, lowering the lung defenses and weakening resistance to pathogen colonization and the development of secondary bacterial infection [66,67,68]. These effects could be explained if we consider the damage of the cellular and intestinal barrier junctions caused by viral infection, cytokines, and dysbiosis itself, resulting in the translocation of opportunistic pathogens present in the gut in COVID-19 subjects to the blood and lymphatic streams [37,38,39,40]. Therefore, the presence of an inflammatory gut microbial environment could promote this succession of events, result in the dysregulation of uncontrolled innate responses to viral infection, reduce adaptive antiviral immunity, and increase susceptibility to secondary infections and tissue damage, causing severe morbidity and mortality in the course of respiratory infectious diseases.

Interestingly, however, the protective effects of SCFAs can extend to extraintestinal tissues, such as lung tissue. Trompette et al. demonstrated that supplementation with butyrate and a fermentable dietary fiber improved the clinical and survival outcomes of severe viral lung infections in mouse models through several mechanisms, including balancing innate and adaptive immune responses, limiting lung tissue damage by enhancing the CD8+ T-lymphocyte effector function and cellular metabolism, and reducing neutrophil chemotactic CXCL1 secretion and the subsequent attenuation of neutrophil infiltration into the airways [69]. Such protective effects of SCFAs have recently been described in mammals with intranasal SARS-CoV-2 infection, including a reduction in the viral load in the airways and intestine through the downregulation of ACE2 expression and enhanced adaptive immunity against VSV/SARS-CoV-2 chimeras through GRP41 and 43 in male animals [70]. Considering the results obtained from these preclinical studies, SCFAs could represent important bacterial mediators for reducing the magnitude of the risk of SARS-CoV-2 infection and post-infectious consequences. In fact, the correlation found between the absence or underrepresentation of SCFA-producing bacteria in COVID-19 subjects and severe disease outcomes [71] suggests the roles of such effects by allowing SARS-CoV-2 infection to become severely symptomatic.

However, more detailed studies are needed in order to understand the reasons for compromised gut health during COVID-19, especially in the more severe forms of the disease. Currently, the relationship between the human gut microbiota and SARS-CoV-2 remains poorly explored. Studies, to date, have demonstrated the involvement of the gut microbiome in the pathogenesis of COVID-19, finding a direct association between dysbiosis and disease severity.

Overall, we can summarize that the current data indicate a reduction in microbiome richness and diversity in COVID-19 individuals compared with healthy subjects. A condition of dysbiosis persists even after viral clearance, increasing patients’ susceptibility to secondary bacterial infections and/or long-term sequelae associated with the chronification of the pro-inflammatory state of the intestinal microenvironment by enrichment with opportunistic pathogens (e.g., *Collinsella, Streptococcus, Coprobacillus, Erysipeltrichaceae, Clostridium ramnosum, Clostribium Hathaway*, etc.) and the depletion of beneficial and/or SCFA-producing symbionts (e.g., *F. prausntizii, Eubacterium, Bifidobacterium, Bacteroides, Ruminococcus, Roseburium, Lachnospiraceae*, etc.). This dysbiotic state is associated with increased levels of plasma markers and inflammatory cytokines, as well as altered intestinal permeability and bacterial translocation, further exacerbating the SARS-CoV-2-induced host immune inflammatory state and prognosis of the disease. Further comparative and longitudinal studies of larger cohorts are needed in order to document the relationship between the human gut microbiota and the clinical effects of acute infection, which may persist even after viral RNA clearance.

Therefore, this particular pattern of dysbiosis could be a prognostic marker of the symptomatic severity of SARS-CoV-2 infection. This indicates not only the influence of viral infection on the gut microbiome phenotype, but also how a targeted approach to achieving a healthy microbial profile, including preventive measures to address the over-representation of beneficial SCFA-producing commensals through the use of probiotics, dietary modifications, and a reduction in pro-inflammatory states and associated comorbidities, could be a viable therapeutic strategy for COVID-19 and the maintenance of an optimal immune system.

**Table 1:** Studies investigating the association between the gut microbiome and disease severity in COVID-19 patients.

Population and Characteristics	Methods	Main Results	Ref.
62 COVID-19 patients	Next-generation sequencing of the V4 region of the 16S ribosomal RNA gene. Samples from HC, severe COVID-19, and seasonal Flu patients were collected at the first visit to the hospital.	Infected patients showed: in alpha diversity abundance of *Streptococcus*, *Clostridium*, *Lactobacillus*, and *Bifidobacterium* *Bacteroides*, *Roseburia*, *Faecalibacterium*, *Coprococcus*, and *Parabacteroides* sera levels of IL-18	[48]
33 seasonal flu patients (Flu) 40 healthy controls (HC)			
30 COVID-19 positive patients. *(10 patients with comorbidities)*	16S rRNA gene sequencing. Stool samples were collected upon admission.	Compared to controls, COVID-19 patients had lymphocyte counts and levels of IL-6 and TNF-α bacterial diversity, abundance of *Ruminococcaceae* and *Lachnospiraceae* familyies abundance of opportunistic pathogens: *Streptococcus, Rothia, Veillonella*, and *Actinomyces.* A positive correlation was described between PCR levels and opportunistic bacteria.	[49]
2 groups: 30 healthy controls 24 H1N1 flu patients			
15 COVID-19 positive patients	Whole-genome sequencing. Stool samples were collected 2-3 times per week during hospitalization.	An in *Collinsella aerofaciens* was detected in fecal samples, with high infectivity. In contrast, fecal samples with low SARS-CoV-2 infectivity had levels of *Parabacteroides*, *Bacteroides* and *Lachnospiraeae*. *Coprobacillus*, *Clostridium ramosum*, and *C. hathewayi* in the Firmicutes phylum were the best bacteria, showing a positive correlation with COVID-19 severity.	[64]
15 healthy controls			
15 COVID-19 positive patients	Whole-genome sequencing. Samples were collected 2-3 times per week during hospitalization.	COVID-19 patients on admission, compared to healthy controls, had numbers of *Clostridium hathewayi, Actinomyces viscosus*, and *Bacteroides nordii. Alistipes onderdonkii* and *Faecalibacterium prausnitzii* were negatively correlated with severe COVID-19.	[44]
15 healthy controls			
117 patients infected with SARS-CoV-2	16S rRNA gene sequencing of the V3-V4 region.	In total, virus-positive patients showed a: bacterial richness; *Actinobacteria*, *Bifidobacterium*, *Streptococcus*, and *Collinsella* genera; *Proteobacteria, Bacteroidetes*, *Enterobacteriaceae*, and *Bacteroides* genera. Patients with severe COVID-19 exhibited: blood levels of inflammatory markers; CD8+ T cell number; abundance of *F. prausnitzii* and *Roseburia*.	[51]
95 SARS-CoV-2 negative patients			
50 COVID-19 positive patients, symptomatic (mild, moderate, severe) and asymptomatic	Shotgun next-generation sequencing was performed. Samples were collected within 48 h after the onset of symptoms or within one week after positivity.	Gut microbiota of 28 severely symptomatic SARS-CoV-2 patients had bacterial α-diversity, levels of the protective *Bifidobacterium, Faecalibacterium*, and *Roseburium* genera, and abundance of *Bacteroides.*	[54]
20 COVID-19 negative controls; exposed controls			

### 3.3. Probiotic Supplementations: Possible Strategy for Modulating the Gut Microbiota in COVID-19

The use of probiotics as a strategy for modulating the composition of the gut microbiota in COVID-19 patients has not been sufficiently explored. However, evidence has been accumulated to support the beneficial effects of supplementation with probiotics on numerous disease states. Preclinical and clinical studies have documented the positive and immunomodulatory effects of manipulating the gut microbiota against respiratory tract infections following probiotic administration. The beneficial effects of probiotics are believed to be mediated mainly by increasing SCFA production and strengthening gastrointestinal-associated lymphoid tissues (GALT).

In this regard, *Bifidobacterium* ssp. is one of the most frequently used probiotics, being a major component of the microbiota and having important immune functions, such as increasing the Treg responses, reducing cell damage by inhibiting TNF-α and macrophage activity, and protecting against intestinal epithelial cell damage [72]. A randomized trial evaluated the effects of *Bifidobacterium longum* BB536 on diarrhea and/or upper respiratory tract disease in 520 children, showing that the probiotic reduced the duration of common upper respiratory tract infections (URTIs) by modulating the gut microbiota, resulting in increased bacterial genera with immunomodulatory and anti-inflammatory properties, such as Faecalibacterium, in the treated group compared with the placebo group [73]. Improvements in URTIs and influenza virus infections were also obtained following the administration of *Lactobacillus plantarum* DR7, a bacterial strain that acts on the activation of AMP protein kinase (AMPK) and the macrophages [74]. Reduced plasma levels of IFN-γ and TNF-α and increased anti-inflammatory cytokines (IL-4, IL-10) were also observed, accompanied by reduced levels of oxidative stress, the decreased expression of CD4 and CD8 cells, and the increased presence of NK cells in adults who received DR7 compared with the placebo [74]. Similar results have been reported following the oral administration of *Lactobacillus casei* Zhang to act against URTIs and gastrointestinal disorders due to potential antioxidative and immunomodulatory effects [75]. These immune regulatory functions of probiotic strains could play a pivotal role in modulating the immune response, recomposing a healthy gut microbial phenotype, and reducing the risk of beneficial severity during SARS-CoV-2 infection.

To test this hypothesis, a randomized, controlled, single-center, open-label clinical trial evaluated the efficacy of a multi-strain probiotic (*L.rhamnosus, B.bifidum, B.longum, B.infantis*) in the treatment of COVID-19 in 99 hospitalized patients with pneumonia and 101 control patients [76]. The study revealed that the consumption of probiotics had no significant effects on mortality, the total duration of the illness, the incidence of intensive care unit admissions, the need for mechanical ventilation or oxygen support, the development of liver injury, or changes in the levels of most inflammatory biomarkers of COVID-19. However, it was effective in treating COVID-19-associated diarrhea, which resolved sooner in the treated group than in the control group (2 days compared to 4), and in preventing hospital-acquired diarrhea in patients who received only one antibiotic. Another randomized, quadruple-blind study of 300 symptomatic adult patients with COVID-19 disease, assigned (1:1) either a probiotic formula (*Lactiplantibacillus Plantarum* and *Pediococcus acidilactici* strains) or a placebo to the participants for 30 days [77]. The data showed that probiotic supplementation reduced the nasopharyngeal viral load, pulmonary infiltrates, and duration of digestive and non-digestive symptoms compared to placebo. No significant changes in the composition of the fecal microbiota were detected between the two groups, but probiotic supplementation significantly increased the production of specific IgM and IgG against SARS-CoV2 compared to the placebo, suggesting that the probiotic acts directly by interacting with the host immune system rather than by changing the composition of the colonic microbiota [77].

According to ClinicalTrials.gov, 11 clinical trials on the effects of probiotics on COVID-19 infection have been completed (ClinicalTrials.gov Identifier: NCT04621071, NCT05474144, NCT04390477, NCT04937556, NCT04734886, NCT05043376, NCT05175833, NCT04847349, NCT04462627, NCT04517422, NCT04399252). Another 14 are still ongoing, and the results may provide future directions for the prevention of this disease.

## 4. Nutrition and COVID-19: An Evidence-Based Overview of Risk Factors and Protective Factors

### 4.1. Implications of Metabolic Alterations and Malnutrition

Severe disease progression and mortality rates for COVID-19 are highly dependent on several factors, such as age, pre-existing comorbidities, the speed of treatment implementation, and responses to therapy.

During the global health emergency, the National Health Service (NHS) classified the risk factors for critical SARS-CoV-2 disease as high or moderate, identifying oncology and transplant patients and subjects with severe lung disease as ‘high risk’. Other important risk factors for severe complications and increased mortality in COVID-19 patients are age >70 years, obesity (BMI ≥ 30), cardiovascular disease, and metabolic syndrome (MetS) [78]. In fact, according to clinical observations, 99% of deaths in Italy due to severe COVID-19 occurred in elderly, overweight, or obese individuals affected by hypertension, type-II diabetes and insulin resistance, or cardiovascular diseases [79,80], indicating that metabolic alterations may perform a relevant role in the clinical manifestation of COVID-19 infection. Based on this evidence, another European prospective study that included a population of 4000 patients with severe COVID-19 infection also confirmed these data, reporting a 90-day mortality rate of 31% for the most vulnerable population with severe ARDS [81].

The ACE-2 receptor not only allows the coronavirus to bind and enter the target cells but is also involved in insulin resistance in severe COVID-19 patients. ACE2 is involved in the ACE–Ang-II–AT^1^R axis and the ACE-2–Ang-1-7–Mas axis of the RAAS system [82], whose over-regulation and under-regulation, respectively, occur in metabolic disorders and also with age [82]. Physiologically, ACE-2 catalyzes the conversion of angiotensin II to angiotensin (1–7), with vasodilator-related, anti-inflammatory, and antifibrotic effects on the respiratory system, in addition to inducing antioxidant stress [82], protecting against the effects of excessive activation of the ACE–Ang-II–AT^1^R axis [82], with proinflammatory and profibrotic effects in the respiratory system, and causing vascular dysfunction, myocardial fibrosis, nephropathy, and RAAS insulin resistance [82]. Therefore, under normal conditions, we can observe the control of the cardiovascular risk, a reduction in cellular oxidative stress, and an improvement in insulin signaling.

SARS-CoV-2 infection causes the upregulation of the ACE2 receptor expression in the lungs and kidneys, as demonstrated in K18-hACE2 mice who underwent intranasal inoculation with standard SARS-CoV-2 [61]. Normal levels of ACE2 are necessary in order to combat inflammatory lung disease [83]. However, it has been observed that the baseline expression of ACE-2 is increased in many diseases, such as lung cancer [61], and in patients with diabetes [80]. A further marked upregulation of ACE2 has been identified in COVID-19 patients with chronic pulmonary comorbidities, pulmonary fibrosis, asthma, lung cancer, chronic obstructive pulmonary disease (COPD), diabetes, and hypertension [61]. This suggests that, in elderly patients and those with metabolic disorders such as obesity, hyperglycemia, insulin resistance, or MetS, who already have a compromised innate and humoral immune system due to their comorbidities, ACE-2 over-expression may contribute to an increased susceptibility to SARS-CoV-2 infection and the severe course of disease [80].

In this context, clinical reports show that malnourished obese subjects infected with SARS-CoV-2 have a poor prognosis, mainly due to a pre-existing low-grade inflammatory state characterized by high concentrations of TNF-α, MCP-1, and IL-6, which are chronically produced by visceral adipose tissue and innate immunity and could promote the triggering of an uncontrolled inflammatory reaction (cytokine storm) following viral infection [84,85]. Such pro-inflammatory effects may be further marked if we consider the involvement of the gut microbiome in metabolic and immune homeostasis. Indeed, several authors have indicated the presence of gut dysbiosis in metabolic disorders [86,87], proposing that an altered host–bacteria interaction may cause intestinal permeability dysfunction and facilitate the systemic translocation of bacterial LPS, contributing to metabolic endotoxemia, as well as the development of severe liver and lung inflammation in preclinical models of COVID-19 [86]. In addition, obesity increases the pressure in the pleural cavity by reducing pulmonary recruitment and alveolar collapse, resulting in organ compromise.

On the opposite end of the metabolic spectrum, malnutrition is also a short- and long-term prognostic factor for increased mortality, especially in older patients with restricted food intake. Interestingly, mortality rates due to COVID-19 are much lower in East Asian rice-consuming countries than in other wheat-consuming countries of the world [88]. Inadequate diet, dehydration, and poor nutritional intake, whether general or individual nutrients, alter the cellular metabolism and immune system function, increasing the risk of severe complications of SARS-CoV-2 infection. Indeed, many hospitalized patients, including those with COVID-19, have a poor nutritional status on admission due to a loss of appetite, dysphagia, shortness of breath, and reduced consciousness [89]. In addition, reduced levels of lymphocytes, albumin, and pre-albumin (indicators of nutritional screening) in association with inflammation-induced hyper-catabolism, fever, swallowing problems, and increased work of the respiratory muscles in pneumonia patients further elevate the risk of mortality and a worse prognosis [90]. Not surprisingly, according to the ESPEN guidelines for the intensive care unit [91], early nutritional support may be implemented in order to maintain an adequate macronutrient and energy intake and meet the recommended daily allowance (RDA) for vitamins and minerals due to their anti-inflammatory and antioxidant potential. Therefore, assessing the nutritional risks of patients with COVID-19, especially the elderly, should always be a well-performed hospital procedure.

### 4.2. Role of Nutrients and Bioactive Components

Several molecules affect the immune and inflammatory response, influencing the number and functions of immune cells through direct effects on the signal transduction pathways responsible for cell metabolism. In particular, nutrition plays a considerable role in immunity, as certain nutrients have an immunomodulatory action and promote the balance between the formation of pro- and anti-inflammatory mediators. To help to strengthen this positive effect, the well-known Mediterranean dietary regimen recommends that people should consume at least five portions of fruit and vegetables per day in their regular diet and involves a higher consumption of whole grains, dried fruit, and extra virgin olive oil, aiming to reduce the risk of developing metabolic and inflammatory diseases. Conversely, a ‘Western diet’ characterized by a higher intake of animal fats, refined cereals, and sugars, should be limited due to its systemic pro-inflammatory effects. These effects are also markedly accentuated by the influences of the two different dietary and lifestyle patterns on the structure and activity of the gut microbiome. Inspiring results have shown that nutrition influences lung immunity [92]. A diet rich in dietary fiber, in addition to changing the gut microbiota and resulting in increased levels of circulating SCFAs, may also affect the pulmonary microbiota and reduce allergic inflammation in the lungs [92]. In this context, a healthy and balanced ‘Mediterranean diet’ may be important for reducing the risk of severe SARS-CoV-2 infection due to the presence of antiviral and immune-stimulating compounds, such as vitamins, minerals, polyunsaturated fatty acids (PUFA), and flavonoids, but also dietary fiber, which has a direct beneficial effect on the gut microbiome and the production of SCFAs, as previously discussed.

The effects of dietary components on COVID-19, particularly on long-term sequelae, should be studied and determined. In this review, we indicate the effects of some nutrients whose immunomodulatory, anti-inflammatory, and antioxidant actions have been proven in the context of different diseases and which might be associated with the severe course of SARS-CoV-2 infection. In this regard, a systematic review showed that an adequate nutritional status and intake of certain nutrients, such as vitamins A, C, D, and E, omega-3 fatty acids, zinc, and iron, have a positive impact on the nutritional status and immune response to viral infections, such as coronavirus [93].

Specifically, vitamin D receptors are present in many immune cells, with direct and indirect immunomodulatory effects. Vitamin D modulates the T-lymphocyte function by helping to suppress the Th1/Th2 response and the production of IL-1β, IL-6, TNF-α, RA, N, KL, COX-2, and pro-inflammatory nitric oxide in favor of the secretion of anti-inflammatory IL-10 [94]. In addition, cholecalciferol acts on antigen-presenting cells, affecting macrophage monocyte differentiation and antimicrobial peptide expression [95]. Hypovitaminosis D, which is globally prevalent, has been associated with an increased risk of severe COVID-19 and also has important repercussions on comorbidities, such as hypertension, hepatic steatosis, hyperuricemia, and diabetes, which negatively impact the clinical course of SARS-CoV-2 infection [96]. In obese subjects, fat-soluble vitamin D is deposited in the adipose tissue with a further reduction in the blood levels, which, as mentioned earlier, further increases the risk of severe COVID-19. Based on the latest scientific reports, oral supplementation with vitamin D may reduce the risk of severe infection and mortality from SARS-CoV-2 [97].

Continuing with the other beneficial components, Vitamin C participates in several physiological processes associated with immunity through its anti-inflammatory and antioxidant power, demonstrating beneficial actions against colds and pneumonia. Moreover, treatment with high doses of vitamin C has been associated with a better prognosis of lung damage in patients with ARDS who were admitted to intensive care units and undergoing mechanical ventilation [98]. Indeed, ascorbic acid helps to reduce the production of reactive oxygen species (ROS) and maintain the integrity of the epithelial barrier. Moreover, its accumulation in the phagocytic cells helps to enhance chemotaxis and phagocytosis, thus inducing microbial death and apoptosis of damaged cells. Finally, it appears to promote the differentiation and proliferation of B- and T-lymphocytes [99]. In addition, it inhibits NLRP3 inflammasome activation and participates in the blood clotting process, an important mechanism of COVID-19 [98,100]. Thus, it is likely that vitamin C affects COVID-19 infection; thus, it is recommended that levels of vitamin C higher than typical daily doses (500–3000 mg) should be taken during acute viral infection.

Vitamin E acts as an antioxidant and protects cell membranes from tissue damage caused by chain reactions of ROS. In essence, it exists in the form of tocopherols, found in high concentrations in extra virgin olive oil, vegetable oils, nuts, and tocotrienols, which are mainly contained in cereals and some seeds [101]. Animal studies have shown that this vitamin is involved in immunity and host susceptibility to infection, and tocopherol supplementation can increase the resistance to viral infections [102]. Clinical reports state that vitamin E reduces the production of prostaglandin E2 (PGE2) via the inhibition of cyclooxygenase-2 (COX-2) activity, modulating the balance of the Th1/Th2 immune response, and appears to be involved in NK cell cytotoxic activity [101].

Vitamin A also has antioxidant power and modulates adaptive immunity. Among the different functions, it ensures the regeneration of mucous membranes damaged by infection, plays a role in the development of T- and B-lymphocytes, and supports the protective functions of the macrophages, neutrophils, and NK cells. Furthermore, vitamin A may prevent the secretion of cytokines IL-12, preventing the activation of the Th1 response in favor of Th2 [103].

Metals, such as zinc and iron, are found in many foods, including meat, fish, nuts, and legumes. This element has an immunological antioxidant role, in addition to influencing adiposity and insulin resistance, which may aggravate metabolic dysregulation, contributing to an inadequate inflammatory response [104]. Furthermore, a study showed the inhibitory effect of high Zn2+ concentrations in mitigating the activity of the coronavirus RNA-dependent RNA polymerase (RdRp) (SARS-CoV nsp12) [105]. The immunomodulatory role of iron has also been widely confirmed. It is, indeed, involved in the regulation of the differentiation and proliferation of T-helper lymphocytes and cytotoxic T-lymphocytes, as well as the pro-inflammatory M1 response. In addition, zinc plays a role in the production of IFN-γ and cytokines, as well as in stimulating the production of ROS by neutrophils involved in the killing of pathogens [106].

Additional scientific evidence supports the important roles of dietary components and metabolites derived from the activity of the gut microbiota, with beneficial downstream effects on immune homeostasis and inflammation. The main metabolites presenting protective mechanisms are SCFAs, ω-3 fatty acids, and flavonoids.

SCFAs, which include acetate, butyrate, and propionate, are short-chain fatty acids that are produced by saccharolytic bacteria belonging to the gut microbiota, such as Lactobacilli and Bifidobacteria, following the ingestion of non-digestible dietary carbohydrates. Several studies have emphasized the health benefits of dietary fiber, including its effects on the GI tract (i.e., the prevention of damage caused by pathogens, improvement of the gut barrier function, and modulation of the immune system and gut microbiota) and on the cardiovascular and metabolic system (i.e., the reduction in blood lipid levels and insulin resistance) [107], and the improvement of the lung function and reduction in mortality rates due to respiratory diseases. Therefore, plant-based diets, functional foods, and supplements represent a promising strategy for protection against respiratory infections. From an immunomodulatory perspective, SCFAs facilitate immunological tolerance to food antigens, promote Treg cell responses, and contribute to the maintenance of intestinal integrity by involving the NALP3 and IL-18 inflammasome pathway [108]. Furthermore, SCFAs have multiple anti-inflammatory effects on leukocyte recruitment and nuclear factor (NF)-κB inhibition. More specifically, SCFAs have been reported to reduce the expression of vascular cell adhesion proteins and intracellular adhesion molecules, the production of inflammatory chemokines, and the production of TNF-α, IL-6, and IFN-γ [108,109].

A deficiency in omega-3 essential fatty acids, mainly eicosapentaenoic acid (EPA) and docosahexaenoic acid (DHA), may also increase one’s susceptibility to viral diseases [110]. Their role is to inhibit pro-inflammatory processes and suppress the immune response. However, their immunomodulatory activity is strictly dependent on the omega-3/omega-6 dietary intake ratio, which should be 1:5 [111]. In addition, they are involved in promoting Treg cell proliferation, inhibiting neutrophil and monocyte activation, and priming the activity of nuclear transcription factors that promote the transcription of genes encoding for TNF-α, IL-1β, and IL-6 [112,113]. A recent pilot study reported that lower circulating blood levels of omega-3 fatty acids were associated with a four-fold increased risk of death from COVID-19, suggesting that the intake of DHA and EPA might help the immune system to fight SARS-CoV-2 infection and improve symptoms in infected patients [114]. Recently, Chiang et al. stated that N-3 PUFAs, mainly DHA and EPA, can be considered as preventive agents that block SARS-CoV-2 infection [115]. In conclusion, the authors suggested that the anti-infection effects of N-3 PUFAs are associated with the inactivation of the NF-κB signaling pathway and the reduced expression of angiotensin-converting enzyme 2 (ACE2) and downstream transmembrane serine protease 2 in human endothelial progenitor cells (hEPCs), following the stimulation of the bacterial metabolite trimethylamine-N-oxide (TMAO) [115].

**Figure 1:**
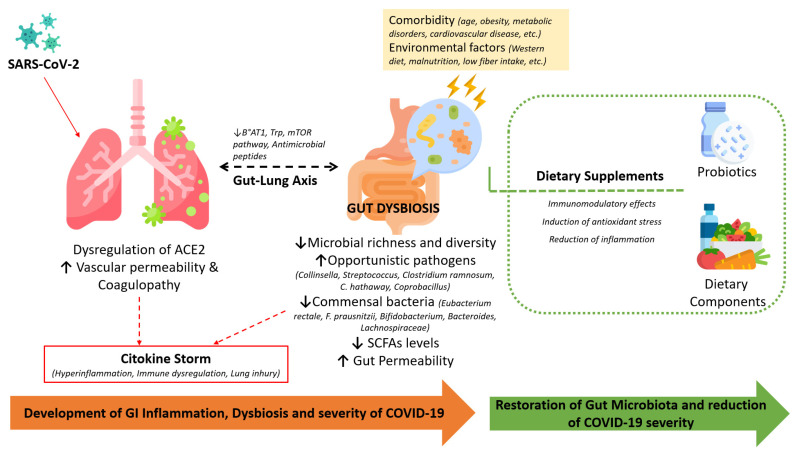
The involvement of the infection-induced dysregulation of ACE-2, comorbidities, and alterations in the gut microbiota during COVID-19 and the beneficial effects of dietary supplements in restoring the microbiota and immune homeostasis.

Figure 1 is a summary diagram showing how SARS-CoV-2 pathogenesis affects the gut–lung axis according to the mechanisms considered in this review.

## 5. Conclusions

The microbiota composition of the GI is involved in shaping the immune response to a variety of pathogens, including SARS-CoV-2. Dysbiosis, both before and during SARS-CoV-2 infection, can affect the disease progression and clinical outcome in a self-reinforcing virtuous cycle. It is thus clear that interventions aiming to re-establish a correct microbiota composition are important for developing a more holistic approach to managing a series of diseases, including COVID-19. Nutrients and bioactive components have been recently proposed to help to manage COVID symptoms due to their potential beneficial effects, while lacking relevant side effects. Using such compounds to maintain a healthy microbiota and complement pharmacological treatments could thus be considered a viable strategy for increasing resistance to COVID-19 and improving the prognosis of infected patients.

## Data Availability

Not applicable.
